# Supplementary material for: Resolving the full spectrum of human genome variation using Linked-Reads
Source: Genome Res. 2019 Apr;29(4):635–45. doi: 10.1101/gr.234443.118 (PMC6442396; doi:10.1101/gr.234443.118)
Supplement: Supplemental Material [file supp_gr.234443.118_Supplemental_longranger-2.2.zip › longranger-2.2.tar.bz2!/lr2.2-public/lib/go/src/code.google.com/vitess/third_party/py/mock-1.0.1.tar.gz!/mock-1.0.1/html/index.html]

Mock - Mocking and Testing Library — Mock 1.0.1 documentation


### Navigation

- index
- next |
- Mock 1.0.1 documentation »

# Mock - Mocking and Testing Library¶

|  |  |
| --- | --- |
| Author: | Michael Foord |
| Version: | 1.0.1 |
| Date: | 2012/10/07 |
| Homepage: | Mock Homepage |
| Download: | Mock on PyPI |
| Documentation: | PDF Documentation |
| License: | BSD License |
| Support: | Mailing list (testing-in-python@lists.idyll.org) |
| Issue tracker: | Google code project |

mock is a library for testing in Python. It allows you to replace parts of
your system under test with mock objects and make assertions about how they
have been used.

mock is now part of the Python standard library, available as unittest.mock
in Python 3.3 onwards.

mock provides a core Mock class removing the need to create a host
of stubs throughout your test suite. After performing an action, you can make
assertions about which methods / attributes were used and arguments they were
called with. You can also specify return values and set needed attributes in
the normal way.

Additionally, mock provides a patch() decorator that handles patching
module and class level attributes within the scope of a test, along with
sentinel for creating unique objects. See the quick guide for
some examples of how to use Mock, MagicMock and
patch().

Mock is very easy to use and is designed for use with
unittest. Mock is based on
the ‘action -> assertion’ pattern instead of ‘record -> replay’ used by many
mocking frameworks.

mock is tested on Python versions 2.4-2.7, Python 3 plus the latest versions of
Jython and PyPy.

## API Documentation¶

- The Mock Class
- Calling
- Deleting Attributes
- Attaching Mocks as Attributes
- Patch Decorators
  - patch
  - patch.object
  - patch.dict
  - patch.multiple
  - patch methods: start and stop
  - TEST\_PREFIX
  - Nesting Patch Decorators
  - Where to patch
  - Patching Descriptors and Proxy Objects
- Helpers
  - call
  - create\_autospec
  - ANY
  - FILTER\_DIR
  - mock\_open
  - Autospeccing
- Sentinel
  - Sentinel Example
- Mocking Magic Methods
- Magic Mock

## User Guide¶

- Getting Started with Mock
  - Using Mock
  - Patch Decorators
- Further Examples
  - Mocking chained calls
  - Partial mocking
  - Mocking a Generator Method
  - Applying the same patch to every test method
  - Mocking Unbound Methods
  - Checking multiple calls with mock
  - Coping with mutable arguments
  - Raising exceptions on attribute access
  - Multiple calls with different effects
  - Nesting Patches
  - Mocking a dictionary with MagicMock
  - Mock subclasses and their attributes
  - Mocking imports with patch.dict
  - Tracking order of calls and less verbose call assertions
  - More complex argument matching
  - Less verbose configuration of mock objects
  - Matching any argument in assertions
  - Mocking Properties
  - Mocking open
  - Mocks without some attributes
- Mock Library Comparison
  - Simple fake object
  - Simple mock
  - Creating partial mocks
  - Ensure calls are made in specific order
  - Raising exceptions
  - Override new instances of a class
  - Call the same method multiple times
  - Mock chained methods
  - Mocking a context manager
  - Mocking the builtin open used as a context manager
- CHANGELOG
  - 2012/11/5 Version 1.0.1
  - 2012/10/07 Version 1.0.0
  - 2012/07/13 Version 1.0.0 beta 1
  - 2012/05/04 Version 1.0.0 alpha 2
  - 2012/03/25 Version 1.0.0 alpha 1
  - 2012/02/13 Version 0.8.0
  - 2012/01/10 Version 0.8.0 release candidate 2
  - 2011/12/29 Version 0.8.0 release candidate 1
  - 2011/10/09 Version 0.8.0 beta 4
  - 2011/08/15 Version 0.8.0 beta 3
  - 2011/08/05 Version 0.8.0 beta 2
  - 2011/07/25 Version 0.8.0 beta 1
  - 2011/07/16 Version 0.8.0 alpha 2
  - 2011/06/14 Version 0.8.0 alpha 1
  - 2011/05/30 Version 0.7.2
  - 2011/05/06 Version 0.7.1
  - 2011/03/05 Version 0.7.0
  - 2011/02/16 Version 0.7.0 RC 1
  - 2010/11/12 Version 0.7.0 beta 4
  - 2010/09/18 Version 0.7.0 beta 3
  - 2010/06/23 Version 0.7.0 beta 2
  - 2010/06/22 Version 0.7.0 beta 1
  - 2009/08/22 Version 0.6.0
  - 2009/04/17 Version 0.5.0
  - 2008/10/12 Version 0.4.0
  - 2007/12/03 Version 0.3.1
  - 2007/11/30 Version 0.3.0
  - 2007/11/21 Version 0.2.1
  - 2007/11/20 Version 0.2.0
  - 2007/11/19 Version 0.1.0
- TODO and Limitations

## Installing¶

The current version is 1.0.1. Mock is stable and widely used. If you do
find any bugs, or have suggestions for improvements / extensions
then please contact us.

- mock on PyPI
- mock documentation as PDF
- Google Code Home & Mercurial Repository

You can checkout the latest development version from the Google Code Mercurial
repository with the following command:

> hg clone https://mock.googlecode.com/hg/ mock

If you have pip, setuptools or distribute you can install mock with:

> easy\_install -U mock
>
> pip install -U mock

Alternatively you can download the mock distribution from PyPI and after
unpacking run:

> python setup.py install

## Quick Guide¶

Mock and MagicMock objects create all attributes and
methods as you access them and store details of how they have been used. You
can configure them, to specify return values or limit what attributes are
available, and then make assertions about how they have been used:

```
>>> from mock import MagicMock
>>> thing = ProductionClass()
>>> thing.method = MagicMock(return_value=3)
>>> thing.method(3, 4, 5, key='value')
3
>>> thing.method.assert_called_with(3, 4, 5, key='value')
```

side\_effect allows you to perform side effects, including raising an
exception when a mock is called:

```
>>> mock = Mock(side_effect=KeyError('foo'))
>>> mock()
Traceback (most recent call last):
 ...
KeyError: 'foo'

>>> values = {'a': 1, 'b': 2, 'c': 3}
>>> def side_effect(arg):
...     return values[arg]
...
>>> mock.side_effect = side_effect
>>> mock('a'), mock('b'), mock('c')
(1, 2, 3)
>>> mock.side_effect = [5, 4, 3, 2, 1]
>>> mock(), mock(), mock()
(5, 4, 3)
```

Mock has many other ways you can configure it and control its behaviour. For
example the spec argument configures the mock to take its specification
from another object. Attempting to access attributes or methods on the mock
that don’t exist on the spec will fail with an AttributeError.

The patch() decorator / context manager makes it easy to mock classes or
objects in a module under test. The object you specify will be replaced with a
mock (or other object) during the test and restored when the test ends:

```
>>> from mock import patch
>>> @patch('module.ClassName2')
... @patch('module.ClassName1')
... def test(MockClass1, MockClass2):
...     module.ClassName1()
...     module.ClassName2()

...     assert MockClass1 is module.ClassName1
...     assert MockClass2 is module.ClassName2
...     assert MockClass1.called
...     assert MockClass2.called
...
>>> test()
```

Note

When you nest patch decorators the mocks are passed in to the decorated
function in the same order they applied (the normal *python* order that
decorators are applied). This means from the bottom up, so in the example
above the mock for module.ClassName1 is passed in first.

With patch it matters that you patch objects in the namespace where they
are looked up. This is normally straightforward, but for a quick guide
read *where to patch*.

As well as a decorator patch can be used as a context manager in a with
statement:

```
>>> with patch.object(ProductionClass, 'method', return_value=None) as mock_method:
...     thing = ProductionClass()
...     thing.method(1, 2, 3)
...
>>> mock_method.assert_called_once_with(1, 2, 3)
```

There is also patch.dict() for setting values in a dictionary just
during a scope and restoring the dictionary to its original state when the test
ends:

```
>>> foo = {'key': 'value'}
>>> original = foo.copy()
>>> with patch.dict(foo, {'newkey': 'newvalue'}, clear=True):
...     assert foo == {'newkey': 'newvalue'}
...
>>> assert foo == original
```

Mock supports the mocking of Python *magic methods*. The
easiest way of using magic methods is with the MagicMock class. It
allows you to do things like:

```
>>> mock = MagicMock()
>>> mock.__str__.return_value = 'foobarbaz'
>>> str(mock)
'foobarbaz'
>>> mock.__str__.assert_called_with()
```

Mock allows you to assign functions (or other Mock instances) to magic methods
and they will be called appropriately. The MagicMock class is just a Mock
variant that has all of the magic methods pre-created for you (well, all the
useful ones anyway).

The following is an example of using magic methods with the ordinary Mock
class:

```
>>> mock = Mock()
>>> mock.__str__ = Mock(return_value='wheeeeee')
>>> str(mock)
'wheeeeee'
```

For ensuring that the mock objects in your tests have the same api as the
objects they are replacing, you can use *auto-speccing*.
Auto-speccing can be done through the autospec argument to patch, or the
create\_autospec() function. Auto-speccing creates mock objects that
have the same attributes and methods as the objects they are replacing, and
any functions and methods (including constructors) have the same call
signature as the real object.

This ensures that your mocks will fail in the same way as your production
code if they are used incorrectly:

```
>>> from mock import create_autospec
>>> def function(a, b, c):
...     pass
...
>>> mock_function = create_autospec(function, return_value='fishy')
>>> mock_function(1, 2, 3)
'fishy'
>>> mock_function.assert_called_once_with(1, 2, 3)
>>> mock_function('wrong arguments')
Traceback (most recent call last):
 ...
TypeError: <lambda>() takes exactly 3 arguments (1 given)
```

create\_autospec can also be used on classes, where it copies the signature of
the \_\_init\_\_ method, and on callable objects where it copies the signature of
the \_\_call\_\_ method.

## References¶

Articles, blog entries and other stuff related to testing with Mock:

- Imposing a No DB Discipline on Django unit tests
- mock-django: tools for mocking the Django ORM and models
- PyCon 2011 Video: Testing with mock
- Mock objects in Python
- Python: Injecting Mock Objects for Powerful Testing
- Python Mock: How to assert a substring of logger output
- Mocking Django
- Mocking dates and other classes that can’t be modified
- Mock recipes
- Mockity mock mock - some love for the mock module
- Coverage and Mock (with django)
- Python Unit Testing with Mock
- Getting started with Python Mock
- Smart Parameter Checks with mock
- Python mock testing techniques and tools
- How To Test Django Template Tags
- A presentation on Unit Testing with Mock
- Mocking with Django and Google AppEngine

## Tests¶

Mock uses unittest2 for its own
test suite. In order to run it, use the unit2 script that comes with
unittest2 module on a checkout of the source repository:

> unit2 discover

If you have setuptools as well as
unittest2 you can run:

> python setup.py test

On Python 3.2 you can use unittest module from the standard library.

> python3.2 -m unittest discover

On Python 3 the tests for unicode are skipped as they are not relevant. On
Python 2.4 tests that use the with statements are skipped as the with statement
is invalid syntax on Python 2.4.

## Older Versions¶

Documentation for older versions of mock:

- mock 0.8
- mock 0.7
- mock 0.6

Docs from the in-development version of mock can be found at
mock.readthedocs.org.

## Terminology¶

Terminology for objects used to replace other ones can be confusing. Terms
like double, fake, mock, stub, and spy are all used with varying meanings.

In classic mock terminology
mock.Mock is a spy that
allows for *post-mortem* examination. This is what I call the “action ->
assertion” [1] pattern of testing.

I’m not however a fan of this “statically typed mocking terminology”
promulgated by Martin Fowler. It confuses usage
patterns with implementation and prevents you from using natural terminology
when discussing mocking.

I much prefer duck typing, if an object used in your test suite looks like a
mock object and quacks like a mock object then it’s fine to call it a mock, no
matter what the implementation looks like.

This terminology is perhaps more useful in less capable languages where
different usage patterns will *require* different implementations.
mock.Mock() is capable of being used in most of the different roles
described by Fowler, except (annoyingly / frustratingly / ironically) a Mock
itself!

How about a simpler definition: a “mock object” is an object used to replace a
real one in a system under test.

|  |  |
| --- | --- |
| [1] | This pattern is called “AAA” by some members of the testing community; “Arrange - Act - Assert”. |

### Table Of Contents

- Mock - Mocking and Testing Library
  - API Documentation
  - User Guide
  - Installing
  - Quick Guide
  - References
  - Tests
  - Older Versions
  - Terminology

#### Next topic

The Mock Class

### This Page

- Show Source

### Quick search


Enter search terms or a module, class or function name.

### Navigation

- index
- next |
- Mock 1.0.1 documentation »

© Copyright 2007-2012, Michael Foord & the mock team.
Last updated on Nov 05, 2012.
Created using Sphinx 1.1.3.
